# Neurodegenerative disease-associated mutants of a human mitochondrial aminoacyl-tRNA synthetase present individual molecular signatures

**DOI:** 10.1038/srep17332

**Published:** 2015-12-01

**Authors:** Claude Sauter, Bernard Lorber, Agnès Gaudry, Loukmane Karim, Hagen Schwenzer, Frank Wien, Pierre Roblin, Catherine Florentz, Marie Sissler

**Affiliations:** 1 Architecture et Réactivité de l’ARN, CNRS, Université de Strasbourg, IBMC, 15 rue René Descartes, 67084 STRASBOURG Cedex, France; 2 Synchrotron SOLEIL, L’Orme des Merisiers Saint Aubin, 91410 Gif-sur-Yvette, France; 3 URBIA-Nantes, INRA Centre de Nantes, 60 rue de la Géraudière, 44316 Nantes, France

## Abstract

Mutations in human mitochondrial aminoacyl-tRNA synthetases are associated with a variety of neurodegenerative disorders. The effects of these mutations on the structure and function of the enzymes remain to be established. Here, we investigate six mutants of the aspartyl-tRNA synthetase correlated with leukoencephalopathies. Our integrated strategy, combining an ensemble of biochemical and biophysical approaches, reveals that mutants are diversely affected with respect to their solubility in cellular extracts and stability in solution, but not in architecture. Mutations with mild effects on solubility occur in patients as allelic combinations whereas those with strong effects on solubility or on aminoacylation are necessarily associated with a partially functional allele. The fact that all mutations show individual molecular and cellular signatures and affect amino acids only conserved in mammals, points towards an alternative function besides aminoacylation.

Mitochondria are the powerhouses of the eukaryotic cell, hosting the production of energy in the form of ATP by oxidative phosphorylation of ADP. They possess their own genome (mt-DNA), which codes in humans for 13 of the respiratory chain subunits, 22 tRNAs and 2 rRNAs. More than one thousand proteins, encoded by the nuclear genome, synthesized in the cytosol and subsequently imported into mitochondria, are also required for mitochondrial biogenesis and functioning (reviewed in e.g.^1^). Defects in mt-DNA were correlated to human disorders more than 25 years ago^2^. More recently, mitochondrial disorders were also associated with mutations within imported molecules, like factors of the mitochondrial translation machinery^3^,^4^,^5^. Among these are mitochondrial aminoacyl-tRNA synthetases (mt-aaRSs), which catalyze the esterification of tRNAs with cognate amino acids, and are key actors of the synthesis of the 13 respiratory chain subunits.

In 2007, it was shown for the first time that a mt-aaRS is implicated in a disease when mutations were found in the *DARS2* gene coding for the mitochondrial aspartyl-tRNA synthetase (mt-AspRS)^6^. This attracted the attention of the medical community and the steadily growing number of cases reported since then led to the current statement that all mt-aaRS genes (except *WARS2*) are affected by pathology-related mutations^1^,^7^,^8^,^9^,^10^,^11^,^12^. Mt-aaRSs are impacted in various ways, despite being ubiquitously expressed and having a common role in protein biosynthesis. Mutations cause an unexpected variety of phenotypic expressions, including mainly neurological disorders but also non-neurological symptoms in some cases. In every case, however, they have tissue-specific phenotypic imprints. The cause of this selective vulnerability is not understood (reviewed in e.g.^1^,^5^,^8^,^13^), and neither is the way mutations affect the structure and/or function of mt-aaRSs.

Human mt-AspRS is the most prominent case with the largest number of reported mutations (60). These are spread throughout the *DARS2* gene within a cohort of 78 patients belonging to 58 families^6^,^14^,^15^,^16^,^17^,^18^,^19^. All these mutations are correlated with Leukoencephalopathy with Brain stem and Spinal cord involvement and Lactate elevation (LBSL), an autosomal recessive neurological disorder most often manifesting in early childhood and showing a wide spectrum of clinical phenotypes^20^. LBSL patients suffer from impairment of motor function and manual ability and loss of cognitive function to various degrees. Magnetic resonance imaging reveals characteristic abnormalities in the cerebral white matter, the cerebellum, brainstem, and spinal cord. In general, if the first neurological signs of disease appear in infancy (infantile/early onset LBSL), they are followed by severe neurological deterioration and the loss of the ability to walk in adolescence. If symptoms appear later (adult/late onset LBSL), the disease evolves at a slower pace but the patients need support for walking or become wheelchair dependent at the end of their lives (reviewed in^19^). LBSL patients are mainly compound heterozygous^19^, but cases of homozygous mutations have been described in two families^17^,^21^. More than two thirds of the compound heterozygotes carry a mutation in a poly-pyrimidine tract at the 3′ end of intron 2 in one allele that affects the correct splicing of the third exon. This leads to a frameshift and a premature stop, but the defect is called “leaky” because it does not fully stop the expression of full-length mt-AspRS^6^.

Initial *in vitro* analyses of an initial set of human mt-AspRS mutants revealed that only some of them had lower aminoacylation activities^22^,^23^ suggesting that the protein synthesis housekeeping function is not a general target of the mutations. Further investigation showed that any step in the life of the enzyme can be impacted, as shown for pre-mRNA splicing^24^, protein expression and dimerization^23^, and protein translocation from the cytosol into mitochondria^25^. However, the link between the consequence of a mutation at the protein level and the observed clinical phenotype remains unclear.

We recently determined the X-ray structure of human mt-AspRS^26^. This class II homodimeric synthetase shares a common architecture with its bacterial homologs, but is more thermolabile and exhibits a higher plasticity for the binding of tRNA^26^. Based on this knowledge we selected six clinically relevant single point mutations (Fig. 1) located in the N-terminal anticodon-binding domain (R58G, T136S), in the catalytic domain (Q184K, R263Q) or in the C-terminal extension (L613F, L626Q). Phenotypes of corresponding patients are listed in Supplementary Table 1. Previous *in vitro* assays had shown that R263Q is the only mutation of these six that significantly impairs the aminoacylation property of recombinant protein^23^. To investigate if and how these mutations change the solubility, thermal stability and structure in solution of mt-AspRS, we applied an integrated strategy based on comparative biochemical and biophysical analyses. The implications of these observations are discussed in the context of LBSL clinical pictures.

## Results

**Figure 1:**
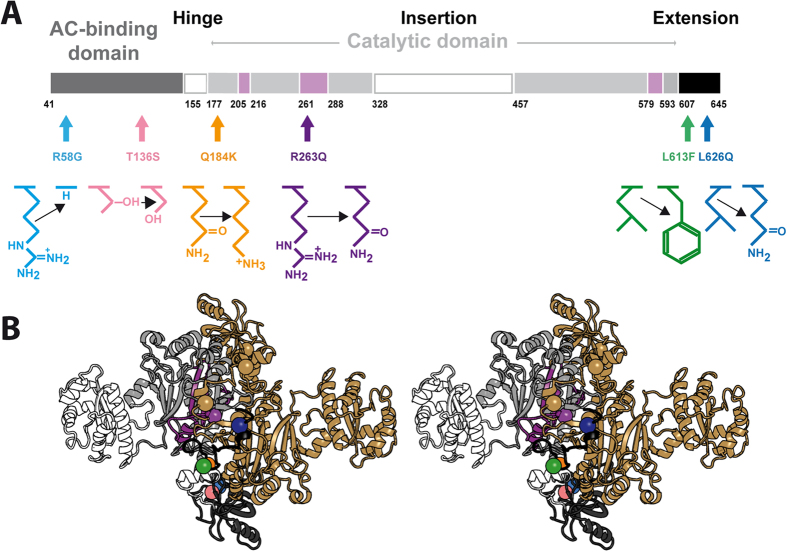
Locations and chemical nature of the amino acid substitutions of LBSL-associated mutations within functional domains of mt-AspRS. (**A**) Schematic representation of the modular organization of the human mt-AspRS (adapted from^23^). Structural modules are the anticodon (AC) binding domain (dark grey), the hinge region (white), and the catalytic domain (light grey). –Insertion– and –Extension– stands for bacterial-type insertion (white) and C-terminal extension (black) domains, respectively. Numbers correspond to amino acid positions flanking the different domains. Pink boxes situate catalytic motifs 1, 2, and 3, specific from class II aaRSs^48^. (**B**) Stereo view of the crystal structure of mt-AspRS (PDBid: 4AH6) with the positions of the studied mutations highlighted by color spheres. Color code on one monomer is as in panel (**A**). The second monomer is shown in gold. All molecular representations were prepared with PyMOL (Schrödinger, Inc.).

In the present study, wild-type (WT) mt-AspRS and six mutants (Fig. 1) were produced in *E. coli* for *in vitro* biophysical analysis. Proteins were expressed without the first 40 N-terminal amino-acids, corresponding to the putative MTS, and with a His-tag appended at the C-terminus. After purification as initially reported for the WT enzyme^27^, all were homogeneous according to dynamic light scattering (DLS) and SDS-PAGE criteria, with masses expected from their amino acid compositions. Aminoacylation activities were in agreement with those reported^23^. The same set of WT and mutant mt-AspRS was expressed in mammalian cells for *in cellulo* solubility analysis as full-length sequences flanked by a C-terminal Flag^®^-tag for immunodetection. In this case, the MTS was naturally cleaved after import into mitochondria.

For both *in vitro* and *in cellulo* experiments, the mutants and the WT synthetase were prepared in parallel and treated in the same way for strictly comparative biochemical and biophysical analyses of solubility (by protein quantification), thermal stability (by DLS; differential scanning fluorimetry, DSF; and synchrotron radiation circular dichroism, SRCD), 2D and 3D structure in solution (by SRCD and small angle X-ray scattering, SAXS). The stabilizing effect of a synthetic inhibitor derived from the aspartyl-adenylate (Asp-AMS), which binds to the active site of the enzyme^26^, was also assessed by DSF.

### Structural properties of recombinant proteins in solution

**Figure 2:**
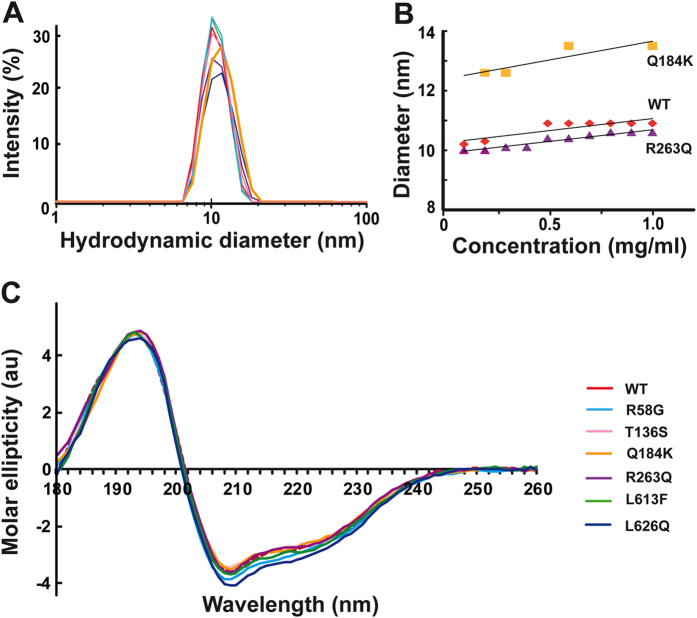
Comparative size and secondary structure elements of WT and mutant mt-AspRSs. (**A**) Particle hydrodynamic diameter distribution by intensity as measured by DLS. (**B**) Concentration dependence of particle diameter of WT, Q184K and R263Q as determined by DLS. (**C**) Overlay of the SRCD spectra of WT and mutants at 24°C. Color code is the same in all panels, as indicated in panel (**C**).

Particle size distributions by intensity derived from DLS measurements performed independently in two instruments indicated that WT and mutants had mean hydrodynamic diameters of d_h_ = 10 ± 1 nm, except Q184K whose size was slightly greater (Fig. 2A and Supplementary Fig. 1). Concentration dependence analysis confirmed that the d_h_ of this mutant was ~12 ± 1 nm after extrapolation to zero concentration (Fig. 2B). SLS analyses also showed that Q184K was distinguished from other samples by a ~20% higher mean molecular mass (results not shown). The content of secondary structure elements in all seven proteins was investigated by SRCD. Far-UV spectra indicated that all contain α-helices, ß-sheets and less structured regions in similar proportions (Fig. 2C, and Supplementary Fig. 2 and Table 2).

### Thermal stability

The stability of WT and mutant proteins was compared by heating them gradually from 24 °C to 80 °C and monitoring either their aggregation by DLS, the binding of a fluorescent dye by DSF, or the effect on their secondary structures by SRCD (Supplementary Fig. 3). The temperatures at which aggregation started in DLS were 40 ± 1 °C for the WT mt-AspRS, 41 ± 1 °C for mutant R263Q, 39 ± 1 °C for mutants R58G, T136S, L613F and L626Q, and 38 ± 1 °C for mutant Q184K. Visual inspection revealed that at the end of these experiments the solutions of mutants Q184K and R263Q had the appearance of boiled egg-white while those of all other proteins contained a layer of aggregated material at the bottom of the DLS cells (results not shown).

**Figure 3:**
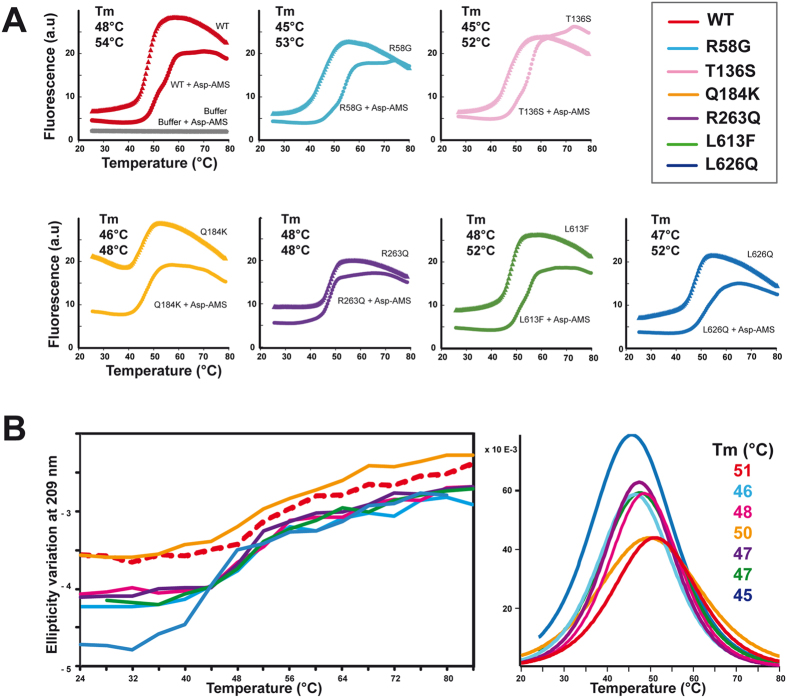
Differential thermal stability of WT mt-AspRS and mutants. (**A**) Temperature-dependent binding of a fluorescent hydrophobic dye measured by DSF, in the absence and in the presence of an aminoacyl-adenylate analog 5′-O-[N-(L-aspartyl)sulfamoyl]adenosine (Asp-AMS). (**B**) Thermal stability of structural secondary elements. (Left) Temperature dependent variation of the molar ellipticity at 209 nm measured by SRCD. (Right) Derivatives of sigmoidal fits of curves displayed in the left panel with maxima corresponding to the Tm. Color code is as in Fig. 1.

In DSF experiment, the transition mid-point temperatures derived from increase of fluorescence intensity of R263Q, L613F, and L626Q were comparable to that of WT mt-AspRS (Tm = 47 to 48 ± 1 °C) (Fig. 3A). The Tm of Q184K was lower by ~2 °C and those of R58G and T136S lower by as much as 3 °C. In the presence of Asp-AMS, the Tm values of WT and R58G were 54 ± 1 °C and 53 ± 1 °C, respectively. Those of T136S, L613F, and L626Q were 52 ± 1 °C. The stabilizing effect of Asp-AMS on WT mt-AspRS^26^ was also strong on R58G and T136S (ΔTm = +7 to +8 °C), but lower on L613F and L626Q (ΔTm = +4 to +5 °C), very low on Q184K (ΔTm = +2 °C), and negligible on R263Q (ΔTm = 0 °C) (Fig. 3A).

The structural stability of WT and mutant mt-AspRSs was further characterized by recording SRCD spectra as a function of the temperature to monitor the loss of secondary structure elements during unfolding. CD spectra are displayed in Supplementary Fig. 3. The Tm derived from the variation of ellipticity at 209 nm wavelength corresponding to CD minimum of the native state (Fig. 3B) was 51 °C for the WT, 50° for Q184K, 48 °C for T136S, 47 °C for R263Q and L613F, 46 °C for R58G, and 45 °C for L626Q. These last two mutants were most affected with Tm values up to 5 to 6 °C below that of the WT enzyme.

### Solubility of mt-AspRS mutants *in cellulo*

**Figure 4:**
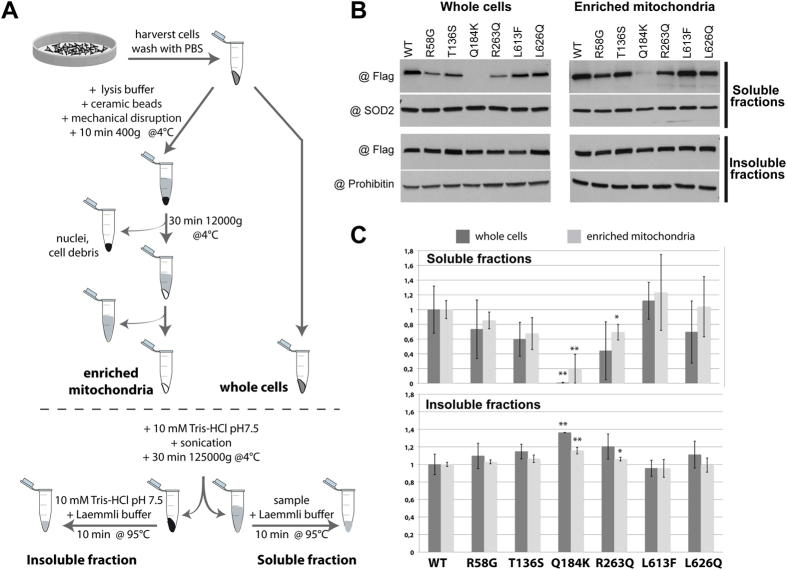
Solubility of WT *versus* mutant mt-AspRSs *in cellulo*. (**A**) Flowchart of experimental procedure. (**B**) Representative western blots of WT and mutant mt-AspRS (detected with anti-Flag antibodies) in soluble and insoluble fractions, within whole cells or enriched mitochondria. Detections of SOD2 (a mitochondrial matrix protein) and prohibitin (a mitochondrial membrane protein) were performed as loading controls for the soluble and the insoluble fractions, respectively. Three sets of independent experiments for whole cells and three sets of independent experiments for enriched mitochondria were analyzed. (**C**) Relative abundance of mutant proteins as compared to WT mt-AspRS in soluble (top) and insoluble (bottom) fractions. Relative amounts of polypeptides were estimated as described in the methods section and normalized according to the WT mt-AspRS, arbitrarily set to a value of 1 in both soluble and insoluble fractions. Errors bars illustrate the standard deviations calculated from the three sets of independent experiments. *p < 0.05 and **p ≤ 0.01 based on Student’s *t*-test.

WT and mutants of mt-AspRS were expressed in BHK21 cells and their abundance in soluble and insoluble fraction in either whole cell extracts or enriched mitochondria was determined by western blot (Fig. 4B). Histograms displaying relative amounts of mt-AspRS in the various fractions are shown in Fig. 4C. Similar trends were found in whole cell extracts and enriched mitochondria: mutant L613F is slightly more soluble than the WT mt-AspRS, L626Q is not significantly impacted, and R58G, T136S and R263Q are slightly less soluble. The most statistically significant decrease in solubility (with p-values ≤ 0.01) was only observed for mutant Q184K.

### Solution structure of mutants Q184K and R263Q *versus* WT

**Figure 5:**
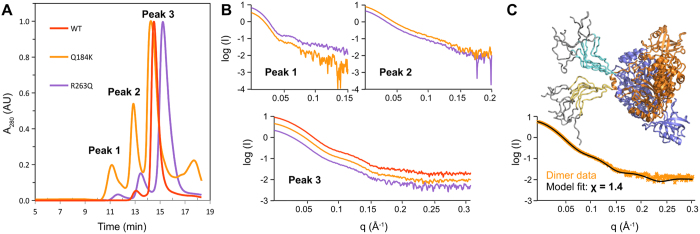
Comparison of WT, Q184K and R263Q mt-AspRSs by SAXS. (**A**) Overlay of normalized SEC absorbance profiles and SAXS curves recorded for each absorbance peak. The WT protein (plots in red) forms essentially a dimer present in peak 3 (93% of the sample), while mutants Q184K (in orange) and R263Q (in violet) also form entities of higher molecular sizes that elute faster in peak 1 (11%, 2% of overall population, respectively) and peak 2 (23%, 9%, respectively). (**B**) SAXS profiles of AspRS populations in peaks 1, 2 and 3. For clarity of presentation, the curves are offset. (**C**) Modeling of mt-AspRS dimer in peak 3 by atomic models generated from the X-ray structure of mt-AspRS (PDBid: 4AH6) to which flexible C-terminal tails were added. In this example, data from Q184K mutant are represented as orange crosses and a typical profile calculated from one of conformers generated under SAXS data constrains by DADIMODO is shown in black. Eight conformations of C-terminal extensions with lowest Chi values (see also Supplementary Fig. 5) are depicted: yellow and cyan tails belong to orange and blue subunit, respectively. The gray region corresponds to the tag used for affinity purification.

Mutants Q184K and R263Q, showing respectively the highest impact on protein solubility in cellular extracts (either whole cells or enriched mitochondria) and the highest impact on aminoacylation *in vitro*^23^, were compared to that of the WT enzyme by SAXS analyses. Each sample was subjected to size exclusion chromatography (HPLC-SEC) directly upstream the SAXS cell to separate aggregates of various sizes from oligomers, allowing individual characterization of each species. The WT enzyme eluted from the SEC column essentially as a single population of particles (UV-absorbance peak 3 in Fig. 5A) while both mutants also contained a significant amount of high molecular-weight particles eluting earlier as peaks 1 and 2 (Fig. 5A). The X-ray scattering signal recorded during the elution of these three protein samples revealed that each peak was composed of well-defined particles yielding similar SAXS curves (Fig. 5B).

The major component of the three protein samples eluting as peak 3 consisted of mt-AspRS dimers with an Rg of 40 ± 1 Å, slightly larger than the Rg (36 Å) calculated from the crystal structure (PDBid: 4AH6), which does not include the C-terminal tails. These tails were not visible by X-ray crystallography probably because they do not adopt a fixed position in the crystal packing. In solution, however, their presence was revealed by the maximal interatomic distance (dmax = 150–155 Å) in the distance distribution function p(r) of the dimer (Table 1). Accordingly, we generated a complete atomic model based on the crystal structure with a C-terminal tail added to each monomer. A series of derived models were selected using DADIMODO (see Methods) that fitted well the experimental SAXS data (Fig. 5C) and gave a much better goodness-of-fit than the crystal structure (Supplementary Fig. 5). Figure 5C shows a superposition of the eight best models and illustrates how the 70 Å long tails extend in the solvent. In the AspRS from *E. coli*, the C-terminal tail encompasses only ten residues and is thus slightly shorter. The last five residues are also not visible in the crystal structure of the free enzyme (PDBid: 1C0A), confirming the flexible nature of C-terminal regions in bacterial-type AspRSs.

The particles eluting in peaks 1 and 2 found in the mutants corresponded to high-order assemblies. While *ab initio* bead modeling did not converge to a satisfactory representation of these entities, the similarity of their SAXS profiles clearly indicated that they were not random aggregates. According to their radii of gyration (Rg = 71 Å and 56 Å, respectively) and their Porod molecular volumes, these large complexes correspond to hexamers of dimers in peak 1 and dimers of dimers in peak 2 (Table 1, Supplementary Fig. 4). A comparison of the integration of the absorbance peaks 1, 2, and 3 of mutant Q184K indicated that the two first high-molecular weight assemblies represent ~11 and 23% of the protein respectively (Fig. 5A). This indicates a strong potential for self-assembly and/or aggregation of this mutant. Under the same experimental conditions, the R263Q sample eluted mainly as dimers (peak 3 contained ~88% of the total protein and peaks 2 and 1 only 9% and 2%, respectively). This mutant interacted with the column matrix as revealed by the reproducible slightly retarded elution. Otherwise, the particle sizes in the three peaks matched with those determined for mutant Q184K. Overall, these results correlate well with the greater size observed in DLS and the predominant occurrence of Q184K in the insoluble fraction *in cellulo*.

## Discussion

Previous studies on human mt-AspRS had shown that mutations associated with the LBSL pathology have various impacts on the molecular and cellular properties of the enzyme, but reduce only marginally the catalytic activity of mt-AspRS and are thus not always deleterious to the housekeeping function^22^,^23^,^24^,^25^. Here, we focused on a set of six mutations and explored how they modify the biophysical properties of the mt-AspRS, specifically its 2D and 3D folds and thermal stability, as well as solubility *in vitro* and *in cellulo*. These clinically relevant mutations, distributed throughout the enzyme sequence, are located either in the anticodon-binding domain opposite to the tRNA binding interface (R58G, T136S), in the catalytic domain at the dimer interface (Q184K, R263Q) or in the C-terminal extension (L613F, L626Q) involved in subunit dimerization within bacterial-type AspRSs.

### Mutations in mt-AspRS sequence context

**Figure 6:**
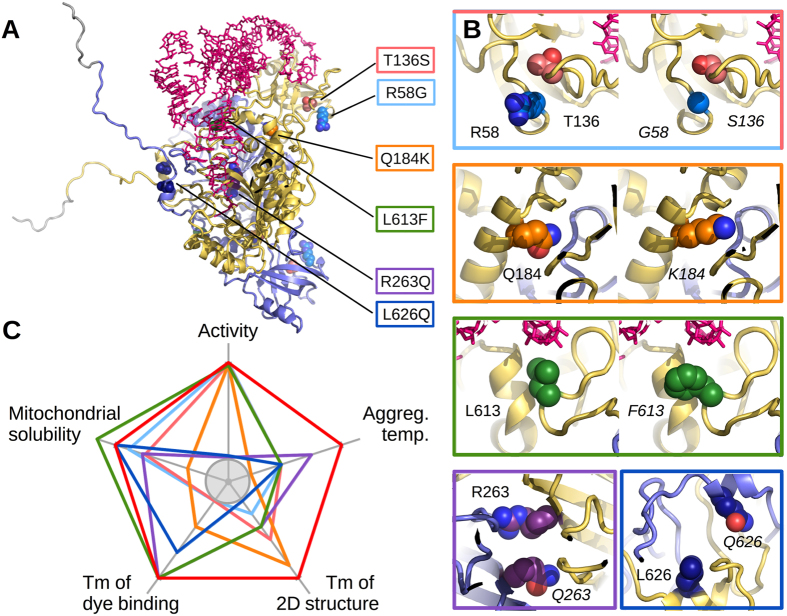
mt-AspRS WT *versus* mutants. (**A**) Positions of single point mutations on the 3D model of mt-AspRS including the two C-terminal extensions and a tRNA molecule docked onto the yellow subunit to show its interaction area (adapted from^26^). (**B**) Close up views showing the original and the mutated side chains in space filling representation. The color of carbon atoms is the same as in other figures. Oxygen and nitrogen atoms depicted in red and blue, respectively. All substitutions are possible without significant steric hindrance. R263Q and L626Q being close to the enzyme 2-fold axis, WT and mutated residues are shown in the same inset. (**C**) Five intrinsic properties ranked relative to the WT (red pentagon = 100% of scale). The enzymatic activity of all mutants was comparable to that of the WT, except that of L626Q and R263Q for which moderate 40 and 140-fold decreases were measured, respectively^23^. Ranges of aggregation temperature determined by DLS, Tm of 2D structure unfolding in SRCD and Tm of dye binding in DSF ranged are 38–41 °C, 45–51 °C and 45–48 °C, respectively. Mitochondrial protein solubilities are taken from top histogram in Fig. 4C. The gray circle delineates the lowest values of each interval. The color code for mutants is indicated. All molecular representations were prepared with PyMOL (Schrödinger, Inc.).

It is widely believed that mutation of strictly conserved residues would be detrimental to the architecture or activity (in the present case, aminoacylation). Therefore we investigated the amino acid conservation of relevant residues in structural sequence alignments made of 180 sequences of bacterial-type AspRSs (60 mitochondrial and 120 bacterial AspRSs). We found that none of them are strictly conserved (Supplementary Table 3), indicating that no gross structural and/or functional perturbations are likely to occur. As seen in Fig. 1A, amino acid substitutions either extend, shorten or delete side chains with either a gain or loss of positive charge or of hydrophobicity. Because of changes in size or the introduction of attractive or repulsive interactions, mutations may compact or expand locally the structure. However, homology models based on the X-ray structure of human WT mt-AspRS suggest that these modifications do not perturb the overall 2D and 3D fold (Fig. 6A,B and Supplementary Table 3). This lack of perturbation is clear for T136S, where mutation replaces a side chain with one of similar volume, and for R58G and L613F where side chains are directed toward the solvent. L613F is the only one occurring in the vicinity of the tRNA binding site but the introduction of a phenyl group at this position is possible without clash with the ligand. In the case of Q184K, R263Q and L626Q at the heart of the dimerization interface, the local structure may cope with the new side chains presenting conformers that do not cause any steric hindrance (Supplementary Table 3).

### Mutations do not affect enzyme architecture

The particle diameters determined by DLS showed that the tested mutants were dimers like the WT mt-AspRS, but that mutant Q184K was of slightly greater size. SAXS data confirmed that mutants Q184K and R263Q, like the WT, are predominantly composed of dimers (Fig. 5). The similarity of SRCD spectra indicated that the secondary structure elements composing the six mutants are present in the same proportions as in the WT enzyme. Altogether, these experimental results demonstrate that the polypeptide chains of all mutants are properly folded and that the 3D architecture of mt-AspRS is not significantly altered by these single point mutations. These results are consistent with the moderate impacts on the ability of the mutants to perform tRNA aminoacylation in the test tube^23^.

SAXS analysis further confirmed the floppy nature of the C-terminal tails in the mt-AspRS dimer that were not visible in the X-ray structure. These extensions pointing into the solvent (Figs 5C and 6A) may provide interaction platforms for putative partners linking the enzyme with alternate mitochondrial networks. The mutations R58G and T136S located on the face opposite to that interacting with the tRNAs could also impair the binding of partner molecules onto mt-AspRS.

### Mutants differ in their stability

All enzymes, WT and mutant mt-AspRSs, sharing the same structure were subjected to thermal denaturation in order to detect differences in stability. Three approaches were used to assess the propensity to aggregate (recorded by DLS), to unfold (visualized by SRCD) and to expose hydrophobic regions to the solvent upon unfolding (monitored by DSF). Interestingly, every mutant behaved in a different manner in response to thermal denaturation. This response varied from one analytical method to the other as summarized in Fig. 6C. The common point was that all mutants were more fragile than the WT protein. These observations raised the question of the stability of the mutants in the cellular environment and at body temperature.

Our previous analyses of human WT mt-AspRS had revealed that the synthetic analog of the aminoacylation intermediate aspartyl-adenylate, Asp-AMS, binds cooperatively^28^ and increases the thermal stability of the protein by as much as 6 °C^26^. This family of compounds is commonly used in crystallographic studies to rigidify the region around the catalytic site^29^,^30^. Here, mutants R58G, T136S, L613F and L626Q were stabilized to the same extent as the WT protein, whereas mutants Q184K and R263Q were not stabilized at all by Asp-AMS. The latter two mutations are located at the dimer interface and may thus hinder the allosteric communication between the two active sites of the synthetase dimer.

### *In cellulo* solubility of WT and mutant mt-AspRSs

The differential thermal stability and effect of Asp-AMS led us to examine the stability of the mutants in the mitochondria of mammalian cells. All recombinant proteins produced in *E. coli* were soluble *in vitro* up to at least 10 mg/ml in standard solvent conditions. The situation was different in BHK cells grown at 37 °C. As shown in Fig. 4, WT and mutant mt-AspRSs were present in the soluble fraction of cellular extracts or of purified mitochondria, except mutant Q184K, which was more abundant in the insoluble fractions. The propensity of this mutant to form insoluble material is consistent with the presence of high-molecular weight objects suspected in DLS analyses and visualized in SEC. SAXS analyses confirmed that the particles in SEC peaks 1 and 2 are not random aggregates but have well defined sizes and shapes (Fig. 5 and Supplementary Fig. 4). These entities correspond to various assemblies of mt-AspRS dimers, e.g. dimers and/or hexamers of dimers (Table 1).

### mt-AspRS mutations in LBSL patients

Selected mutations are found in LBSL patients as compound heterozygous states, meaning that patients *a priori* possess a mixture of homo- and hetero-dimers. Here we examined homodimers composed of either two WT or two mutated monomers, which represent two-thirds of the possible combinations (boxed in Supplementary Fig. 6). Although our approach did not consider heterodimers, it sheds light on the correlation between the strength of the impact of a mutation and the expression of a phenotype.

Q184K and R263Q, the two missense mutations that show the strongest impacts on solubility and activity respectively, are each combined in patients with a splicing defect on the second allele (named R76Serfs*5; Supplementary Table 1). The leakiness of the splicing defect allows the expression of a reduced amount of functional WT enzyme, likely sufficient to sustain mitochondrial translation and to compensate for the depletion in soluble mt-AspRS caused by the presence of aggregation-prone mutants. This hypothesis is supported by the description of another patient, whose allelic composition associates the leaky splicing defect (R76Serfs*5) with the nonsense mutation R263X. The latter generates a completely non-functional molecule. Interestingly, patients R76Serfs*5/R263X and R76Serfs*5/R263Q display similar phenotypes and disease courses (Supplementary Table 1).

Additional compound heterozygous states exist in which the allelic composition combines two missense mutations, i.e. R58G/T136S and L613F/L626Q^19^ (Supplementary Table 1). If we consider our homodimeric mutants R58G, T136S, L613F and L626Q individually, their effects *in cellulo* and *in vitro* are relatively mild with regard to solubility, thermal stability and structure in solution. This suggests that their pathogenicity is likely due to the combination of these effects in patient cells, leading to cumulative deficiencies in their mitochondria.

Altogether, the occurrence of LBSL phenotype seems to be related to the amount of soluble AspRS available in the mitochondria. We observe that allelic compositions in patients (with comparable phenotypes and disease courses, Supplementary Table 1) associate either two mutations with mild individual impacts (i.e. R58G/T136S and L613F/L626Q) or a more deleterious with a non-symptomatic one (i.e. R263Q/R76Serfs*5 and Q184K/R76Serfs*5). This indicates that the strength of the impact of a mutation necessarily falls into a narrow window. In the case of LBSL patients, this window starts where the symptoms become detectable and goes up to a level that is still compatible with a life expectancy greater than 20 years. This is not a general trend since mutations identified in other mt-aaRSs can lead to faster disease courses. In the case of mt-ArgRS for instance, mutations cause severe neonatal or early-infantile epileptic encephalopathy^31^. Strikingly, most of those mutations concern residues that are highly conserved throughout phylogeny.

### Conclusions and perspectives

The main results of the present analysis of six LBSL mutants of human mitochondrial aspartyl-tRNA synthetase (mt-AspRS) can be summarized as follows:

- *In vitro*, mutations have diverse effects on protein solubility and stability, but do not affect enzyme architecture.
- *In cellulo*, five mutants R58G, T136S, R263Q, L613F and L626Q are present like the WT in the soluble fraction of cellular extracts, while mutant Q184K is more abundant in the insoluble fractions.
- In patients, four of the mutants with mild effects on solubility occur as heterozygous pairs in allelic compositions. The two mutants with strongest solubility or activity defect are each combined with a splicing defect on the second allele that allows the expression, albeit at reduced levels, of functional WT enzyme.
- Mutated positions are not strictly conserved throughout phylogeny, in agreement with the fact that none of them leads to gross structural and/or functional perturbations. The exception is residue R263, which is mainly but not strictly conserved. Its mutation causes the strongest impact on aminoacylation.

Up to now LBSL is the neurodegenerative disease correlated with the greatest number of mutations in a human mitochondrial aminoacyl-tRNA synthetase, and with the largest cohort of diagnosed patients. All investigations performed thus far on the possible impacts of LBSL-causing mutations on molecular, cellular, and in the present contribution on biophysical properties of the mt-AspRS, highlight that each mutation has an individual signature (as illustrated in Fig. 6C for those examined here). These observations strengthen the need to further explore cellular properties of mt-AspRS and to search for possible alternative functions, beyond mitochondrial translation. For instance, the C-terminal flexible extensions of mt-AspRS observed by SAXS and pointing into the solvent may provide interaction platforms for putative partners connecting the enzyme with alternate mitochondrial networks. The hypothesis of a non-translational role of mt-AspRS is in agreement with the observation that impacted residues are solely conserved within mammals mt-AspRSs indicating a selective pressure restricted to higher eukaryotes (Supplementary Table 3). This observation holds true as well for additional 18 clinically relevant missense mutations, mainly reported since the beginning of the present work (Supplementary Table 4). While a few of them affect positions highly or strictly conserved throughout all phylogeny, all of them affect positions strictly conserved within mammals. Notably, numerous functions besides translation (often qualified as ‘moonlighting activities’) of cytosolic aaRSs have been described during the past decade (e.g.^32^,^33^,^34^). Interestingly, these functions appeared to have emerged from new selective pressures or new architectural elaborations mainly in vertebrates, and have been shown to be involved in autoimmune disorders, cancers and neurological disorders.

## Material and Methods

### Cells, biochemicals and chemicals

Baby Hamster Kidney cells strain 21 (BHK21) (ATCC # CRL-12072) and modified Vaccinia Ankara strain (MVA-EM24) were generous gifts from Robert Drillien (IGBMC, Strasbourg). Bacterial strain Rosetta^TM^2(DE3) was purchased from Merck Millipore, Anti-Flag^®^ antibody and Tris(2-carboxyethyl)phosphine hydrochloride (TCEP) were from Sigma, anti-SOD2 (human) and anti-prohibitin (human) antibodies were from Abcam^®^, goat anti-rabbit secondary antibody conjugated to horseradish peroxidase [HRP] was from BioRad, chemiluminescent detection kit from Pierce (Thermo Scientific), aminoacyl-adenylate analog 5′-O-[N-(L-aspartyl)sulfamoyl]adenosine (Asp-AMS) from Integrated DNA Technologies (Belgium), and SYPRO Orange^TM^ from Molecular Probes Invitrogen. Mini-Protean® TGX Precast polyacrylamid gels, Trans-Blot Turbo system and SEC protein standard (lyophilized mix containing thyroglobulin, bovine γ-globulin, chicken ovalbumin, equine myoglobin and vitamin B12, MW 1,350–670,000–Cat. No. 151–1901) were from BioRad. Arrest^TM^ protease inhibitor cocktail and polyethylenimine (PEI, linear 25 kDa) were purchased from GBiosciences and Polysciences, respectively.

### Production of recombinant WT and mutant mt-AspRSs

The genes for wild-type mt-AspRS and those carrying the mutations 172C > G (R58G), 406A > T (T136S), 550C > A (Q184K), 788G > A (R263Q), 1837C > T (L613F) and 1876T > A (626Q) were cloned in bacterial strain Rosetta^TM^2(DE3) as previously described^23^,^27^. The wild-type mt-AspRS and the six mutants lacked the 40 N-terminal amino acids and had a VMYLE sequence before the C-terminal 6-His tag. They were purified to homogeneity by affinity and size exclusion chromatography as reported for the WT enzyme^26^,^27^.

### Dynamic (DLS) and static light scattering (SLS)

Samples containing 2 mg/ml mt-AspRS in standard buffer (100 mM potassium phosphate pH 7.5, 50 mM KCl, 0.1 mM EDTA, 10 mM β-mercaptoethanol, and 10% v/v glycerol) were ultracentrifuged for 1 h at 45 000 rpm or 100 000 × g. The supernatant was transferred into 2 μl or 20 μl quartz cells for parallel analyzes at 20 °C in Wyatt Technology Nanostar^TM^ and Malvern Zetasizer^TM^ NanoS light scattering instruments, respectively^35^. Hydrodynamic diameters determined by DLS were corrected for solvent refractive index 1.351 and absolute viscosity 1.463 × 10^−3^ Pa.s. Particle masses determined by SLS were corrected for refractive index concentration dependence dn/dc 0.185 ml/g. The behavior of samples subjected to a temperature gradient from 20 °C to 60 °C was monitored by DLS in the Zetasizer. A shift in the autocorrelation function towards greater times was indicative of the onset of aggregation.

### Differential scanning fluorimetry (DSF)

Twenty μl of mt-AspRS solution containing 2 mg/ml were mixed with 0.5 μl dye (SYPRO Orange^TM^ 5000X stock solution diluted 20-fold in dimethylsulfoxide) in 0.2 ml transparent plastic qPCR tubes. Fluorescence variation was monitored along a temperature gradient from 20 to 90 °C using an Agilent Stratagene Mx3005P instrument. The inflection point of the fluorescence intensity *versus* temperature plot was considered to be the transition mid-point (Tm).

### Synchrotron radiation circular dichroism (SRCD)

SRCD spectra were recorded on the DISCO beamline^36^ at synchrotron SOLEIL (Saint-Aubin, France). The experimental setup was calibrated for magnitude and polarization with a 6.1 mg/ml D-10-camphorsulfonic acid solution. Two μl of WT mt-AspRS or mutants solution at 10 mg/ml in 100 mM potassium phosphate pH 7.5, 50 mM KCl, 10% (v/v) glycerol and 1 mM TCEP were transferred in a CaF_2_ cuvette with an optical path of 8 μm. Three spectra from 170 to 280 nm were recorded at temperatures ranging from 24 to 80 °C with 4 °C steps. They were averaged, solvent baseline subtracted, scaled and normalized to molar ellipticities using CDtool^37^. Protein stability was determined by plotting the variation of the CD minimum at 209 nm as a function of temperature. IGOR software (WaveMetrics) was used to model this variation by a sigmoid and the temperature corresponding to the maximum of its derivative defined a structural transition or melting temperature (Tm). Secondary structure content was estimated from SRCD spectra collected at 24 °C using CONTINLL in Dichroweb and SELCON3 in CDPro using the SP175 reference data set^38^,^39^,^40^. A data cutoff at 180 nm was applied based on photomultiplier high-tension viability. Values were compared to those in the X-ray structure of the WT human mt-AspRS (PDBid: 4AH6).

### Small-angle X-ray scattering (SAXS)

SAXS experiments were performed on the SWING beamline^41^ at synchrotron SOLEIL (Saint-Aubin, France). The beam wavelength was λ = 1.033 Å. The 17 × 17 cm^2^ low-noise Aviex CCD detector was positioned at a distance of 2107 mm from the sample with the direct beam off-centered. The resulting exploitable q-range was 0.005–0.35 Å^−1^, where the wave vector q = 4π sin θ/λ and 2θ is the scattering angle.

WT mt-AspRS and mutants Q184K and R263Q at, respectively, 7.4, 10 and 14 mg/ml in 50 mM HEPES-Na pH 7.5, 150 mM NaCl, 10% (v/v) glycerol, 0.1 mM EDTA and 1 mM DTT, were separated by SEC and analyzed by SAXS online. Thus, 45 μl protein solution was loaded onto an Agilent Bio SEC-3 column (300 Å, 4.6 × 300 mm, 3 μm) installed on an Agilent HPLC system and maintained at 15 °C. Proteins were eluted at a flow rate of 0.2 ml/min with a mobile phase containing 100 mM HEPES-Na pH 7.5, 250 mM NaCl, 5% (v/v) glycerol and 1 mM TCEP. The eluate was analyzed by SAXS in a continuous flow capillary cell with a frame duration of 1000 ms at intervals of 500 ms. Data processing, analysis and modeling step were done with PRIMUS and other programs of the ATSAS suite^42^.

Radii of gyration (R_g_) were derived from Guinier approximation and used to estimate the molecular Porod volume. R_g_ was also calculated from the entire scattering pattern using the indirect transform package GNOM^43^, which provides the distance distribution function p(r) of the particle. A dimeric mt-AspRS model including all residues (two polypeptide chains encompassing residues 41–645 plus a linker of 5 residues and a 6-His-tag) were derived from the X-ray structure (PDBid: 4AH6). The C-terminal tails added to the monomers consisted of the last 15 residues not visible in the crystal structure and of the 11 amino acid affinity tag. The conformational space of these tails was modeled under SAXS constraints using DADIMODO^44^, a genetic algorithm-based refinement analysis program. The core of the dimer corresponding to the crystal structure was treated as a rigid body while the conformation of the C-terminal tails was explored by simulated annealing. A set of eight models were selected that best fit the experimental data. The goodness-of-fit was estimated using CRYSOL^45^.

### Analysis of the soluble/insoluble state of mt-AspRS *in cellulo*

WT and mutants constructs were generated by directed mutagenesis from pBCJ749.77 plasmid, derived from described pBCJ739.14^46^ with the sequence coding for the Flag^®^ tag DYKDDDDK added downstream of the coding sequence of the full-length mt-AspRS including the mitochondrial sequence (MTS) (Consensus CDS Gene CCDS1311.1). Constructs were introduced in BHK21 using a protocol adapted from the transfection-infection approach^46^, under conditions where protein expression is moderate. Cells were washed with PBS and infected with modified vaccinia virus expressing IPTG-inducible T7 polymerase, and subsequently transfected. For transfection, plasmid/PEI complexes were prepared in a mass ratio of 1:4.5 and incubated for 15 min at 20 °C. Protein expression was induced by 1 mM IPTG. 36 h post-transfection, ~5.10^6^ cells were harvested, washed with PBS.

A flowchart of the experimental procedure to investigate soluble/insoluble state of the proteins is given in Fig. 4A. For mitochondrial enrichment, cells were mechanically disrupted using 2 mm diameter ceramic beads in a FastPrep-24^TM^ 5G machine (MP biomedicals), in 1.5 ml of an isotonic lysis buffer (220 mM mannitol, 70 mM sucrose, 1 mM EDTA, 1 mM MgCl_2_, 10 mM HEPES-KOH pH 7.4) containing a protease inhibitor cocktail. Mechanically lysed cells were centrifuged 10 min at 400 g (4 °C). The pellet (nuclei and cell debris) was discarded, and the supernatant was centrifuged 10 min at 12 000 g (4 °C) to pellet mitochondria. Whole cells and mitochondria were sonicated (6 × 10 s) on ice in 160 μl of 10 mM Tris-HCl pH 7.5 containing a protease inhibitor cocktail. The lysates were then ultracentrifuged 30 min at 125 000 g (4 °C) and the soluble fractions (supernatants) were separated from the insoluble fractions (pellets). An aliquot of each soluble fraction (16 μl) was withdrawn and added to 4 μl of Laemmli denaturing buffer, heated to 95 °C for 10 min and loaded on a 10% (m/v) polyacrylamide gel. Pellets were solubilized in 160 μl of 10 mM Tris-HCl pH 7.5 to which 40 μl Laemmli buffer was added before heating and 20 μl of this mixture was analyzed on a gel. Proteins were blotted onto a PVDF membrane and recombinant proteins were detected with an antibody specific for the Flag^®^ tag peptide. Chemiluminescent detection was carried out using the Pierce Detection Kit according to manufacturer’s instructions. Exposed films were digitized using Epson Perfection 3490 Photo scanner and band intensities were quantified on images using ImageJ software^47^.

Mt-AspRS in each sample distributed in either soluble or insoluble fraction, the total corresponding to 100%. The blots of these fractions were developed simultaneously in the same bath to allow for strict comparison of protein amounts and determination of relative distribution in both fractions. In that way, the result was independent of expression. The loading controls, SOD2 or prohibitin respectively for soluble or insoluble fractions, were used to adjust the amount of mt-AspRS in every lane of a given blot. Resulting mt-AspRS intensities were converted to percentages of protein amount (WT or mutants) in soluble and insoluble fractions.

Mean values and standard deviations were derived from three independent experiments. Histograms were normalized with regard to WT mt-AspRS to compare the relative abundance of the mutated proteins in whole cell extracts and enriched mitochondria.

## Additional Information

**How to cite this article**: Sauter, C. *et al.* Neurodegenerative disease-associated mutants of a human mitochondrial aminoacyl-tRNA synthetase present individual molecular signatures. *Sci. Rep.* **5**, 17332; doi: 10.1038/srep17332 (2015).

## Supplementary Material

Supplementary Information

## Figures and Tables

**Table 1:** SAXS analysis of WT mt-AspRS and mutants.

Sample	Peak	Rg Guinier (Å)	Rg p(r) (Å)	dmax (Å)	Porod Vol. (Å^3^)	Oligomer
WT	#3	38.5	39.2	150	230.E3	dimer
R263Q	#1	71.1	71.2	220	145.E4	6× dimer
	#2	53.5	55.0	220	430.E3	2× dimer
	#3	38.7	39.3	155	230.E3	dimer
Q184K	#1	70.8	68.5	200	145.E4	6× dimer
	#2	53.7	56.7	220	430.E3	2× dimer
	#3	40.0	41.3	155	230.E3	dimer
