# Supplementary material for: Neurodegenerative disease-associated mutants of a human mitochondrial aminoacyl-tRNA synthetase present individual molecular signatures
Source: Sci Rep. 2015 Dec 1;5:17332. doi: 10.1038/srep17332 (PMC4664897; doi:10.1038/srep17332)
Supplement: Supplementary Information [file srep17332-s1.doc]

**SUPPLEMENTARY MATERIAL**

**Title: Neurodegenerative disease-associated mutants of a human mitochondrial aminoacyl-tRNA synthetase present individual molecular signatures**

**Author list: Claude SAUTER^1†^*, Bernard LORBER^1†^, Agnès GAUDRY^1^, Loukmane KARIM^1^, Hagen SCHWENZER^1#^, Frank WIEN^2^, Pierre ROBLIN^2,3^, Catherine FLORENTZ^1^ and Marie SISSLER^1^***

^1^ Architecture et Réactivité de l’ARN, CNRS, Université de Strasbourg, IBMC, 15 rue René Descartes, 67084 STRASBOURG Cedex, France

^2^ Synchrotron SOLEIL, L'Orme des Merisiers Saint Aubin, 91410 Gif-sur-Yvette, France;

^3^ URBIA-Nantes, INRA Centre de Nantes, 60 rue de la Géraudière, 44316 Nantes, France.


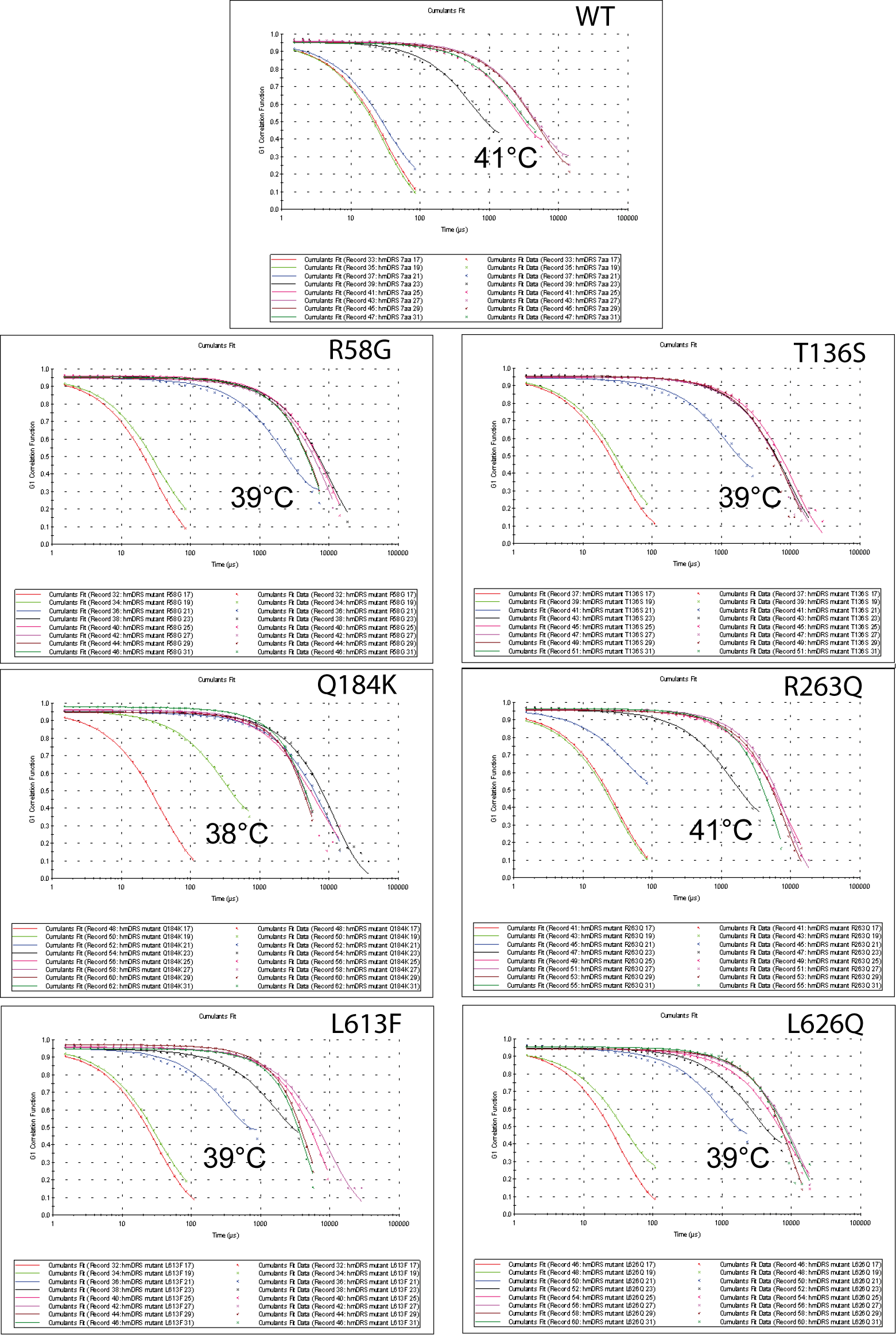


**Supplementary Figure 1: DLS analysis of WT mt-AspRS and mutants as a function of temperature.** A shift in the auto-correlation function indicates a drop in diffusion coefficient (i.e. increase in size). The temperature at which the shift starts is highlighted.


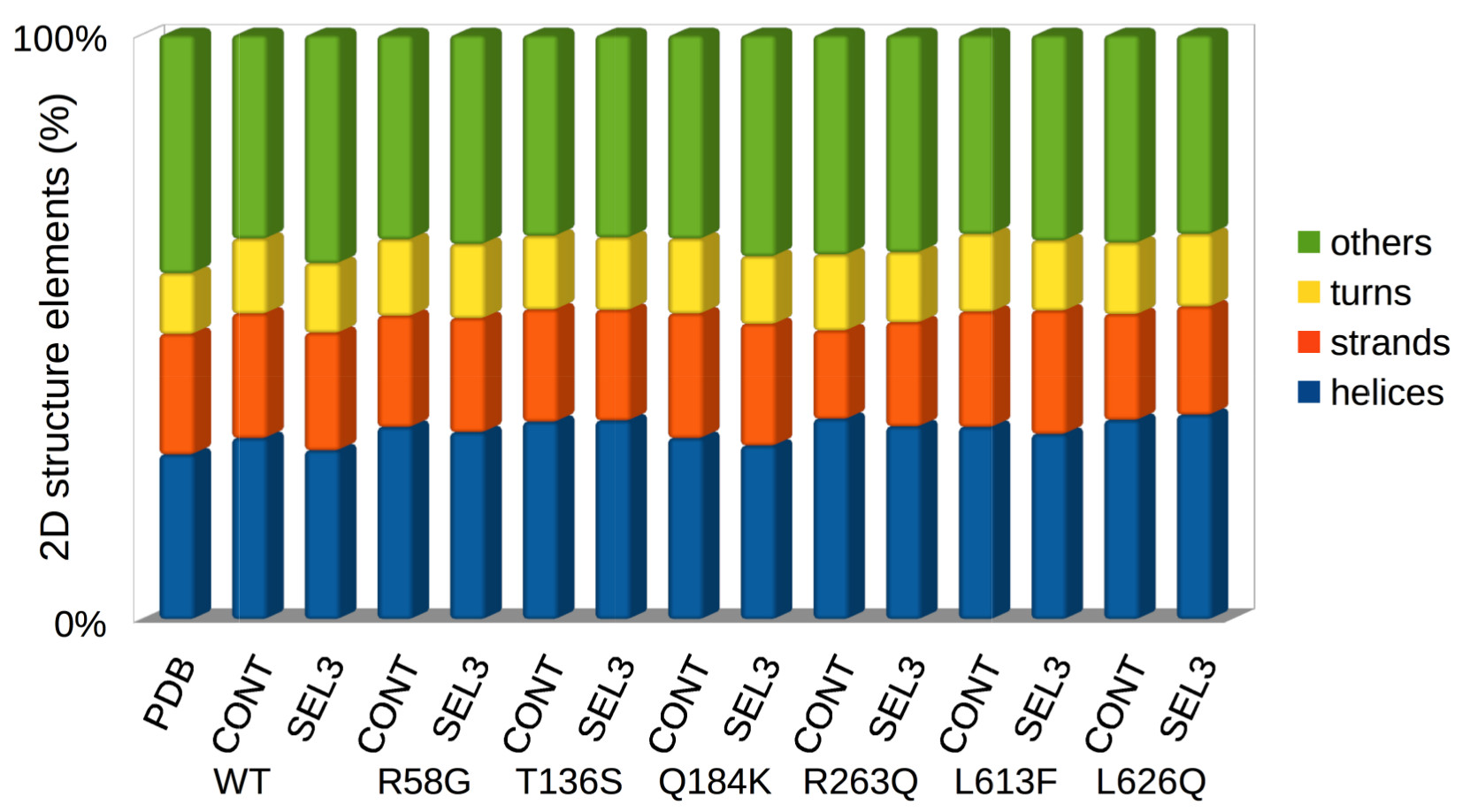


**Supplementary Figure 2: Secondary structure contents of WT mt-AspRS and mutants.** The percentage of helices, beta strands and turns was determined from SRCD spectra collected at 24°C (as displayed in Figure 2) using CONTINLL and SELCON3 (see methods). The distribution of 2D elements in the X-ray structure of mt-AspRS (PDBid: 4AH6) was determined using DSSP^49^. Numerical data are listed in **Supplementary Table 2**.


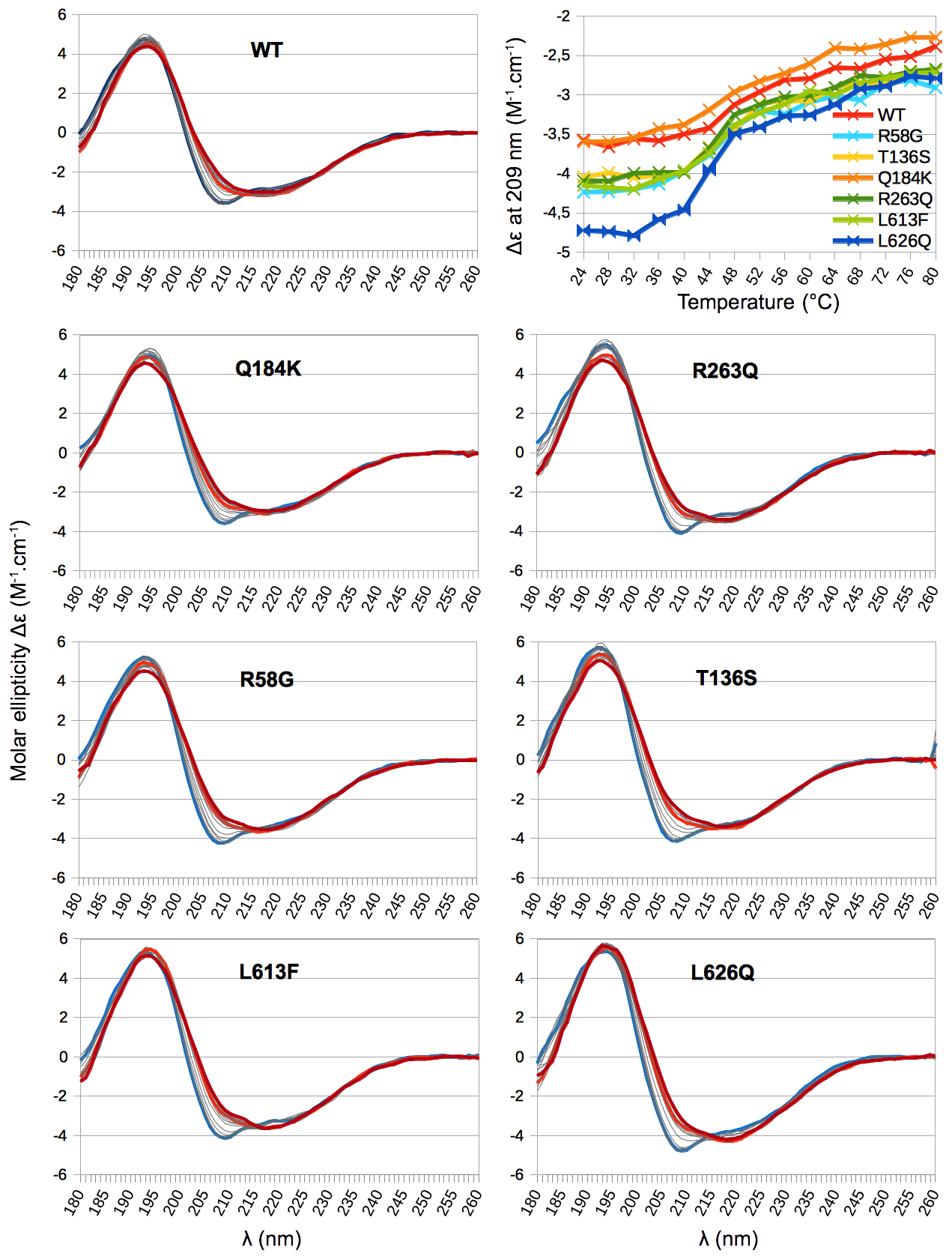


**Supplementary Figure 3: SRCD spectra of WT mt-AspRS and mutants as a function of temperature.** The temperature was stepwise increased from 24°C (blue curve) to 80°C (red curve). (Top right panel) Variation of ellipticity at 209 nm is plotted for each variant as a function of temperature.


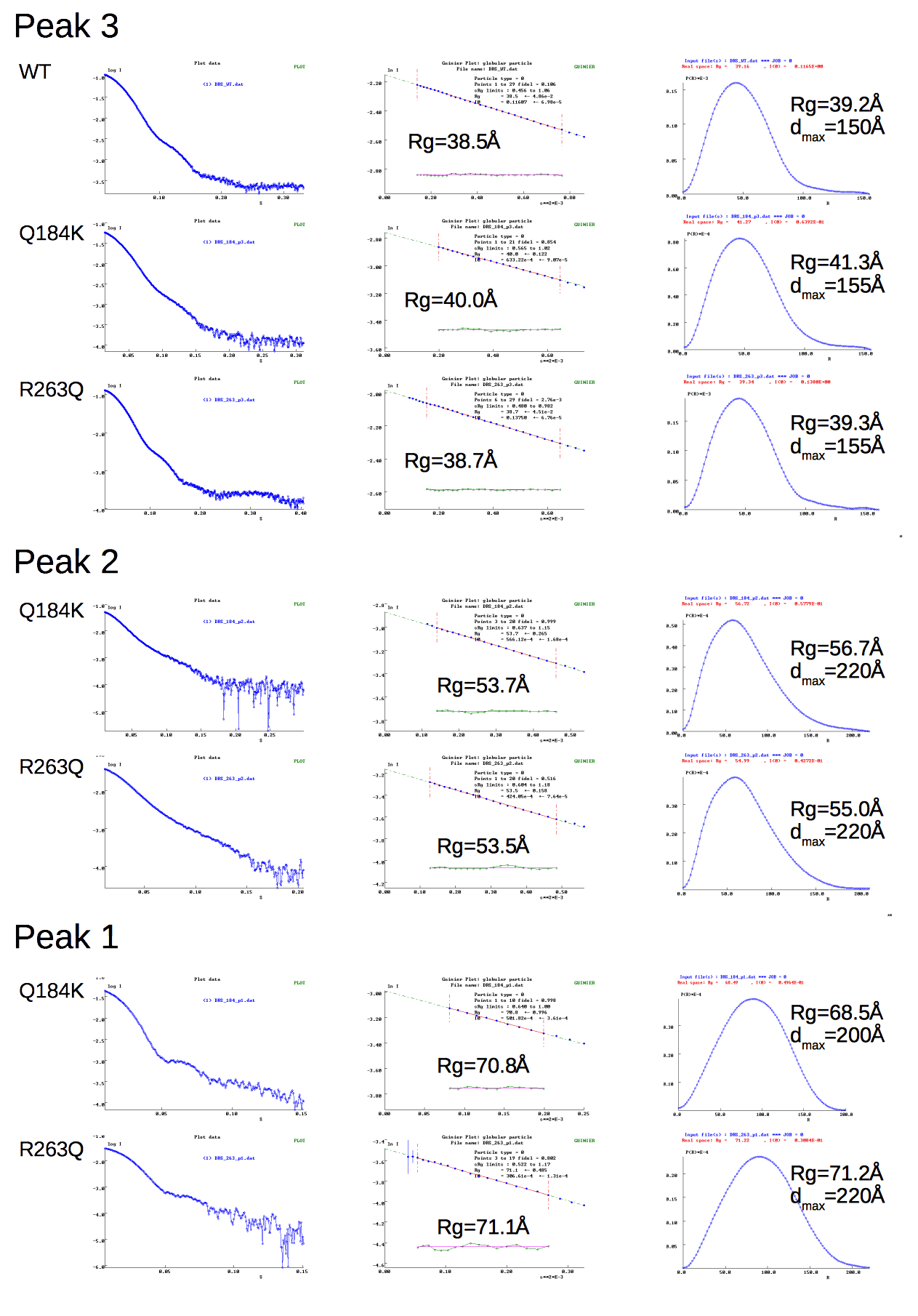


**Supplementary Figure 4: SAXS analysis of WT mt-AspRS and Q184K and R263Q mutants.** (Left, center, right) Experimental SAXS profiles, Guinier plots and P(r) distance distribution functions for each protein population isolated by SEC in peaks 1-3.


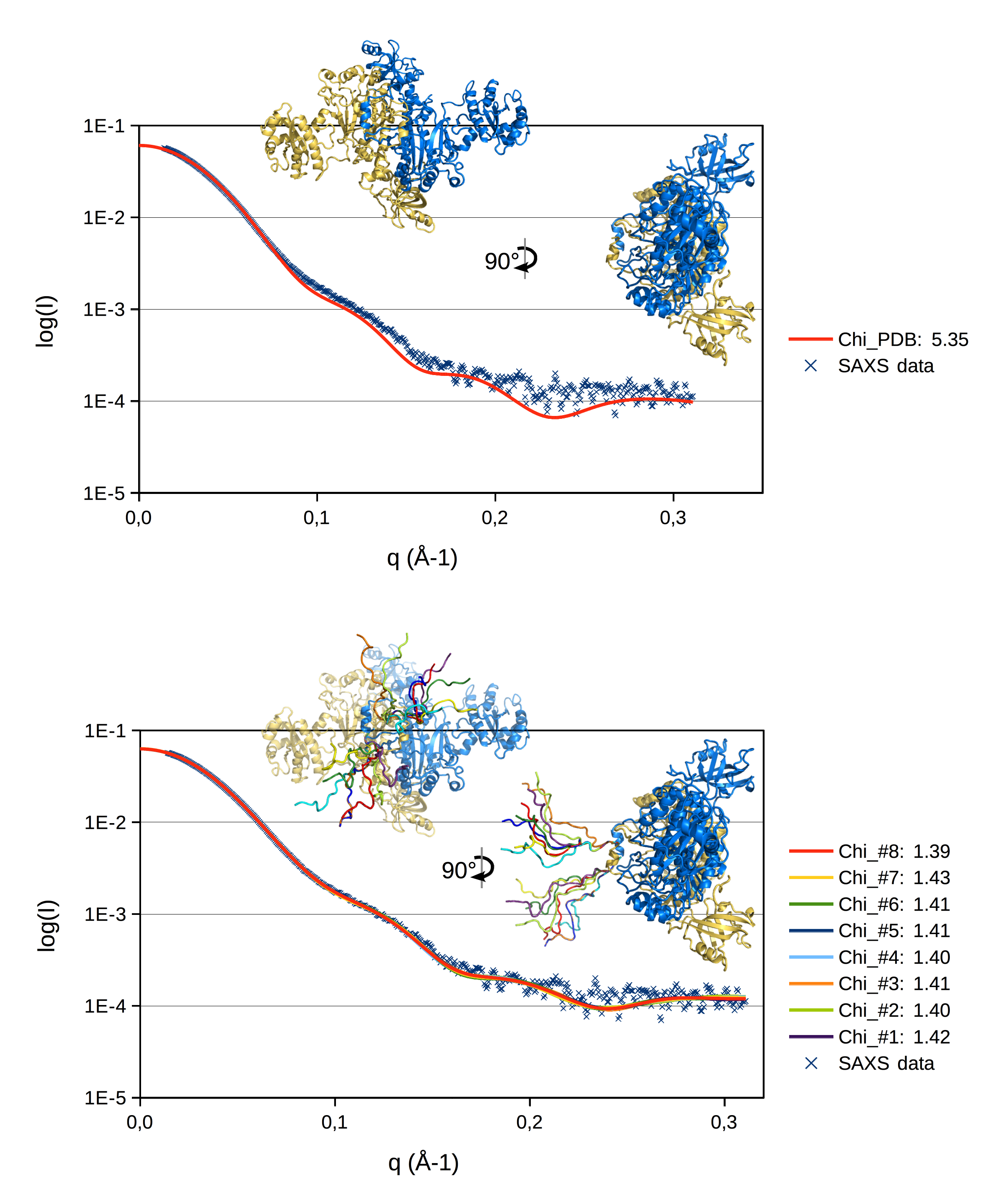


**Supplementary Figure 5: Structure of mt-AspRS dimers in solution.** Experimental SAXS profile of mtAspRS (Q184K mutant - blue dots) was compared to theoretical curves computed for atomic models corresponding to the crystal structure (PDBid: 4AH6) lacking the C-terminal extensions (Top) and to a series of complete models generated by DADIMODO (Bottom). C-terminal extension models and corresponding curves are depicted with the same color. The goodness-of-fit (Chi) was determined using CRYSOL. Models including the C-terminal extensions display a better fit with the data as indicated by lower Chi values.


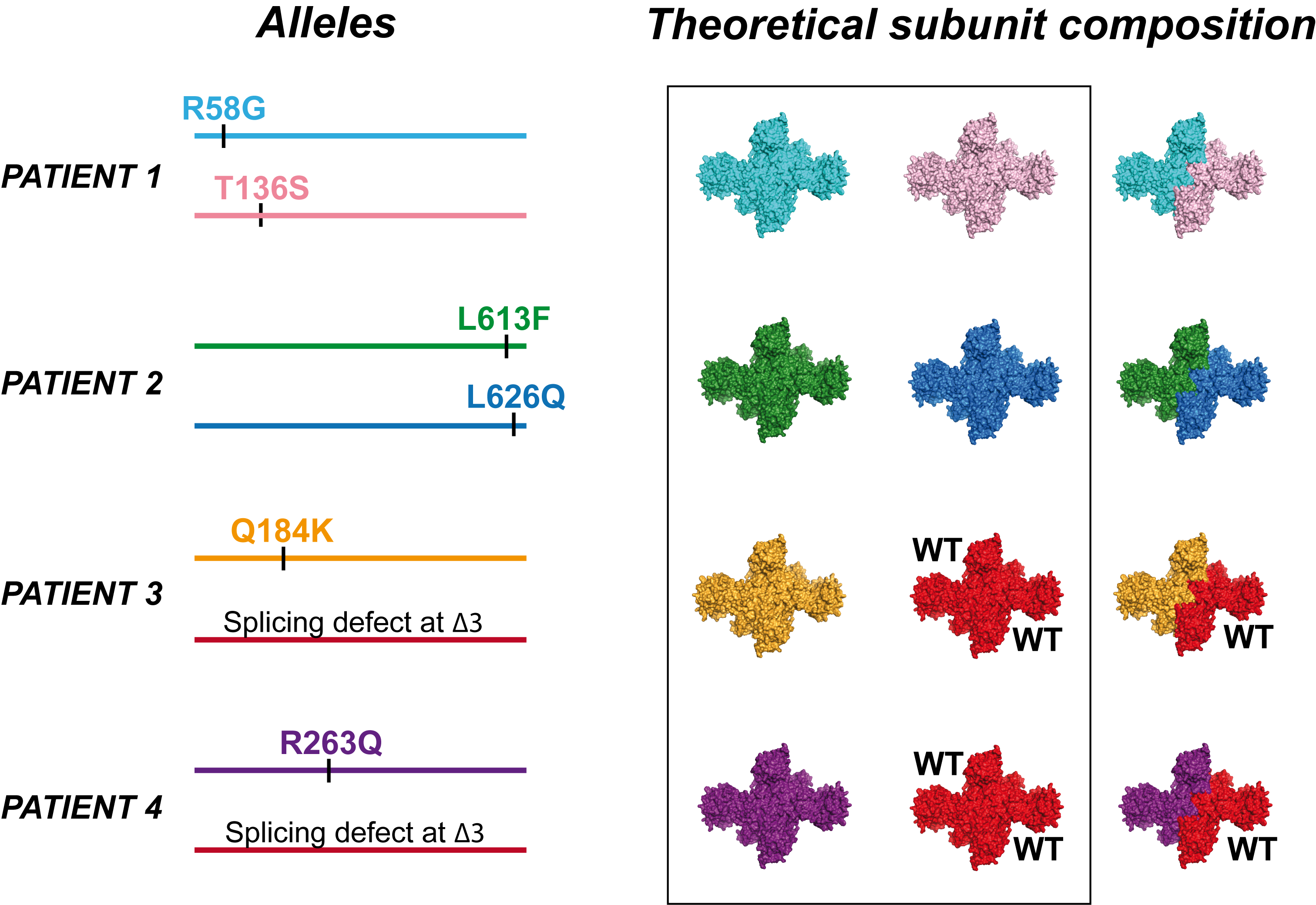


**Supplementary Figure 6:** **Theoretical subunit composition of mt-AspRS dimers resulting from LBSL associated mutations in alleles of heterozygote patients.** Situations explored in this study are boxed.

**Supplementary** Table 1: Phenotypes of LBSL patients with selected mutations

| **Allelic composition** | | **First neurological**  **Signs at years** | **Loss of unsupported walking at years** | **Full wheelchair dependency at years** | **Age in 2013 in years** |
| --- | --- | --- | --- | --- | --- |
| **Mutation 1** | **Mutation 2** |
| R58G | T136S | 3 | - | - | 23 |
| R58G | T136S | 2 | - | - | 15 |
| L613F | L626Q | 12 | 28 | - | 36 |
| Q184K | R76Serfs*5 | 6 | - | - | 22 |
| Q184K | R76Serfs*5 | 7 | 18 | - | 20 |
| R263Q | R76Serfs*5 | 3 | 8 | 20 | 33 |
| R263Q | R76Serfs*5 | 5 | 28 | - | 29 |
| R263X ^a)^ | R76Serfs*5 | 2 | 6 | - | 20 |
| R263X ^a)^ | R76Serfs*5 | 1 | 14 | 22 | 24 |

Adapted from ^50^. ^a)^ not analyzed in the present study but added for comparison.

**Supplementary Table 2: Secondary structure contents derived from SRCD spectra**

**Sample** **WT-mt-AspRS R58G** **T136S** **Q184K R263Q L613F L626Q**

Method PDB CONT / SEL3 CONT / SEL3 CONT / SEL3 CONT / SEL3 CONT / SEL3 CONT / SEL3 CONT / SEL3

Helices 28,2 31,0 / 28,9 32,9 / 32,0 33,8 / 34,0 31,0 / 29,7 34,3 / 33,0 32,9 / 31,7 34,1 / 35,0

Strands 20,6 21,3 / 20,1 19,0 / 19,5 19,2 / 18,9 21,3 / 20,8 15,1 / 17,8 19,7 / 21,1 18,1 / 18,5

Turns 10,4 12,8 / 11,9 13,1 / 12,7 12,6 / 12,4 12,8 / 11,6 13,0 / 12,0 13,3 / 12,0 12,2 / 12,4

Others 40,8 34,9 / 39,1 35,0 / 35,8 34,4 / 34,7 34,9 / 37,9 37,6 / 37,2 34,1 / 35,2 35,6 / 34,1

RMSD 9,8 / 14,6 0,1 / 12,2 13,1 / 12,8 9,8 / 14,6 13,0 / 19,0 12,2 / 13,5 13,9 / 13,4

The percentage of 2D elements was assessed from the PDB entry of WT mt-AspRS using DSSP^49^ and determined from SRCD spectra collected at 24°C using CONTINLL (CONT) and SELCON3 (SEL3). The RMSD of the 2D element estimation is indicated for each method.

**Supplementary** Table 3: Analysis of amino acid conservation and changes in mt-AspRS sequences

| **Mutations** | | **R58G** | **T136S** | **Q184K** | **R263Q** | **L613F** | **L626Q** |
| --- | --- | --- | --- | --- | --- | --- | --- |
| **Location** | | Anticodon-binding domain | Anticodon-binding domain | Catalytic domain | Catalytic domain | C-terminal extension | C-terminal extension |
| **WT residue conservation** | **all seq.** (180) | no | no | no | **R/K** (~97%) | no | no |
|  | **mt mammals** (11) | mainly **R** | yes | yes | yes | only **L/V** | yes |
|  | **mt others** (49) | no | no | no | mainly **R** | **L** (~60%), **P** (~40%) | **L** (~80%), **R** (~20%) |
|  | **bacteria** (120) | no | mainly **T** – a few **S** | no | mainly **R** | **L** (~60%), **P** (~40%) | **L** (~80%), **R** (~20%) |
| **Natural occurrence of substituting residue** | | **G** in some bacteria | **S** in some bacteria | **R/K** in some bacteria; **R** in mt of fungi | **Q** in one bacterium and one mt arthropod | no | no |
| **Structural environment of WT residue** | | Exposed to solvent. No visible interaction with neighbor residues | Hydrophobic environment. Near **L**, **V** and **M** residues | At the beginning of dimerization helix. Involved in a network of hydrogen bonds (G253 from other chain; L178 and R188 of the same chain) | Close to the enzyme 2-fold axis. Interacts with E277 (from same monomer) and T212 (from opposite monomer) | In the vicinity of tRNA binding site | Close to the enzyme 2-fold axis. In the dimerization mini-helix |
| **Theoretical structural impact of the mutation** | | Loss of one positive charge. No local rearrangement | Loss of a methyl group and of van der Waals interactions, but compatible with hydrophobic environment. | 15 out of 18 theoretical conformers lead to steric hindrances. | Loss of a positive charge. Leaves a negative charge with no interactant | 2 out of 4 theoretical conformers lead to steric hindrances. | 4 out of 16 theoretical conformers lead to steric hindrances. |

–Conservation of WT residue– and –Natural occurrence of substitution– were analyzed in a multiple sequence alignment composed of 180 bacterial–type AspRSs, as generated in^51^. This alignment is made of 120 sequences of AspRS proteins from bacteria (encompassing all bacterial subgroups) and 60 sequences of mt-AspRS proteins from eukaryotes (with representatives of mammals, arthropods, fungi and protists). For an objective evaluation of the sequence conservation or divergence, redundancy was avoided by considering only non-identical sequences.

**Supplementary** Table 4: Analysis of amino acid conservation of mt-AspRS positions impacted by disease-associated missense mutations

|  | **Mutations** | **N52S** (ref.^50^) | **R125H** (ref.^50^) | **I139T** (ref.^50^) | **C152F** (ref.^52^) | **R179H** (ref.^52^) | **G206E** (ref.^50^) | **L239P** (ref.^53^) | **Q248K** (ref.^52^) | **L249I** (ref.^54^) |
| --- | --- | --- | --- | --- | --- | --- | --- | --- | --- | --- |
|  | **Location** | anticodon-binding domain | anticodon-binding domain | anticodon-binding domain | anticodon-binding domain | helix of dimerization | catalytic domain (motif 1) | catalytic domain | catalytic domain | catalytic domain |
| **WT residue conservation** | **all seq. (180)** | no | **R/K** (~97%) | no | no | yes (100%) | no | yes (100%) | yes (100%) | no |
|  | **mt mammals (11)** | **yes (100%)** | **yes (100%)** | **yes (100%)** | **yes (100%)** | **yes (100%)** | **yes (100%)** | **yes (100%)** | **yes (100%)** | **yes (100%)** |
|  | **mt others (49)** | no | no (mainly **K**) | no | no | yes (100%) | no | yes (100%) | yes (100%) | no (**L/I/M/T**) |
|  | **bacteria (120)** | no (never a **N**) | yes (100% **R**) | mainly **I/V** | no | yes (100%) | no | yes (100%) | yes (100%) | no (**L/I/M/V**) |
|  | **natural occurrence of substituting res.** | no | no | no | no | no | **E** in some mt others, and in some bacteria | no | no | **I** in some mt others, and in some bacteria |
|  | **allelic composition** | **C152F** | Exon 3 splicing defect | Exon 3 splicing defect | **N52S** | Exon 3 splicing defect | Exon 3 splicing defect | Exon 3 splicing defect | Exon 3 splicing defect | Exon 3 splicing defect |
|  |  |  |  |  |  |  |  |  |  |  |
|  |  |  |  |  |  |  |  |  |  |  |
|  | **Mutations** | **L250P** (ref.^50^) | **G254S** (ref.^50^) | **E284K** (ref.^55^) | **R336H** (ref.^50^) | **P576S** (ref.^50^) | **D560V** (ref.^52^) | **R609W** (ref.^56^) | **L626V** (ref.^52^) | **Y629C** (ref.^52^) |
|  | **Location** | catalytic domain | catalytic domain | catalytic domain (motif 2) | catalytic domain | catalytic domain | catalytic domain | bacterial-type C-terminal extension | bacterial-type C-terminal extension | bacterial-type C-terminal extension |
| **WT residue conservation** | **all seq. (180)** | **L** (~97%) | **G** (~97%) | yes (100%) | **R** (~97%) | yes (100%) | no | no | no | no |
|  | **mt mammals (11)** | **yes (100%)** | **yes (100%)** | **yes (100%)** | **yes (100%)** | **yes (100%)** | **yes (100%)** | **yes (100%)** | **yes (100%)** | **yes (100%)** |
|  | **mt others (49)** | yes (100%) | mainly **G** (some **S/A**) | yes (100%) | mainly **R** (some **C/G**) | yes (100%) | no | no | no | yes (100%) |
|  | **bacteria (120)** | mainly **L** (some **M/C**) | yes (100%) | yes (100%) | yes (100%) | yes (100%) | no | no | no | no |
|  | **natural occurrence of substituting res.** | no | no | no | no | no | no | no | no | no |
|  | **allelic composition** | Exon 3 splicing defect | Exon 3 splicing defect | Exon 3 splicing defect | Exon 3 splicing defect | Exon 3 splicing defect | Exon 3 splicing defect | **R609W** | **L613F** | Exon 3 splicing defect |

Mutations were taken from references ^50, 52-56^ as indicated. They were reported after the beginning of the present work (except for those from ref. ^52^). –Conservation of WT residue– and –Natural occurrence of substitution– were analyzed in a multiple sequence alignment composed of 180 bacterial–type AspRSs, as generated in^51^. This alignment is made of 120 sequences of AspRS proteins from bacteria (encompassing all bacterial subgroups) and 60 sequences of mt-AspRS proteins from eukaryotes (with representatives of mammals, arthropods, fungi and protists). For an objective evaluation of the sequence conservation or divergence, redundancy was avoided by considering only non-identical sequences. Allelic composition recalls the situation found in patients.

**References**

49. Joosten, R.P. et al. A series of PDB related databases for everyday needs. *Nucleic Acids Research* **3**, D411-D419 (2011).

50. van Berge, L. et al. Leukoencephalopathy with brainstem and spinal cord involvement and lactate elevation: clinical and genetic characterization and target for therapy. *Brain* **137**, 1019-1029 (2014).

51. Schwenzer, H. et al. Released selective pressure on a structural domain gives new insights on the functional relaxation of mitochondrial aspartyl-tRNA synthetase. *Biochimie (Special Issue "Mitochondria: an organelle for life")* **100**, 18-26 (2014).

52. Scheper, G.C. et al. Mitochondrial aspartyl-tRNA synthetase deficiency causes leukoencephalopathy with brain stem and spinal cord involvement and lactate elevation. *Nat Genet* **39**, 534-539 (2007).

53. Lin, J. et al. Leukoencephalopathy with brainstem and spinal cord involvement and normal lactate: a new mutation in the DARS2 gene. *J. Child. Neurol.* **25**, 1425-1428 (2010).

54. Labauge, P., Dorboz, I., Eymard-Pierre, E., Dereeper, O. & Boespflug-Tanguy, O. Clinically asymptomatic adult patient with extensive LBSL MRI pattern and DARS2 mutations. *J. Neurol.* **258**, 335-337 (2011).

55. Cheng, F.B. et al. Adult-onset leukoencephalopathy with brain stem and spinal cord involvement in Chinese Han population: a case report and literature review. *Neurol India.* **61**, 161-163 (2013).

56. Synofzik, M. et al. Acetazolamide-responsive exercise-induced episodic ataxia associated with a novel homozygous DARS2 mutation. *J. Med. Genet.* **48**, 713-715 (2011).
